# Supplementary material for: The structural covariance of reading-related brain regions in adults and children with typical or poor reading skills
Source: Dev Cogn Neurosci. 2025 Feb 12;72:101522. doi: 10.1016/j.dcn.2025.101522 (PMC11889628; doi:10.1016/j.dcn.2025.101522)
Supplement: Supplementary file 1 — Supplementary material [file mmc1.docx]

**Supplemental Material**

**Title**

The structural covariance of reading-related brain regions in adults and children with typical or poor reading skills

**Authors**

Amelie Haugg, Nada Frei, Christina Lutz, Sarah V. Di Pietro, Iliana I. Karipidis, Silvia Brem

*Structural covariance matrices in typical-reading adults vs. children*

*
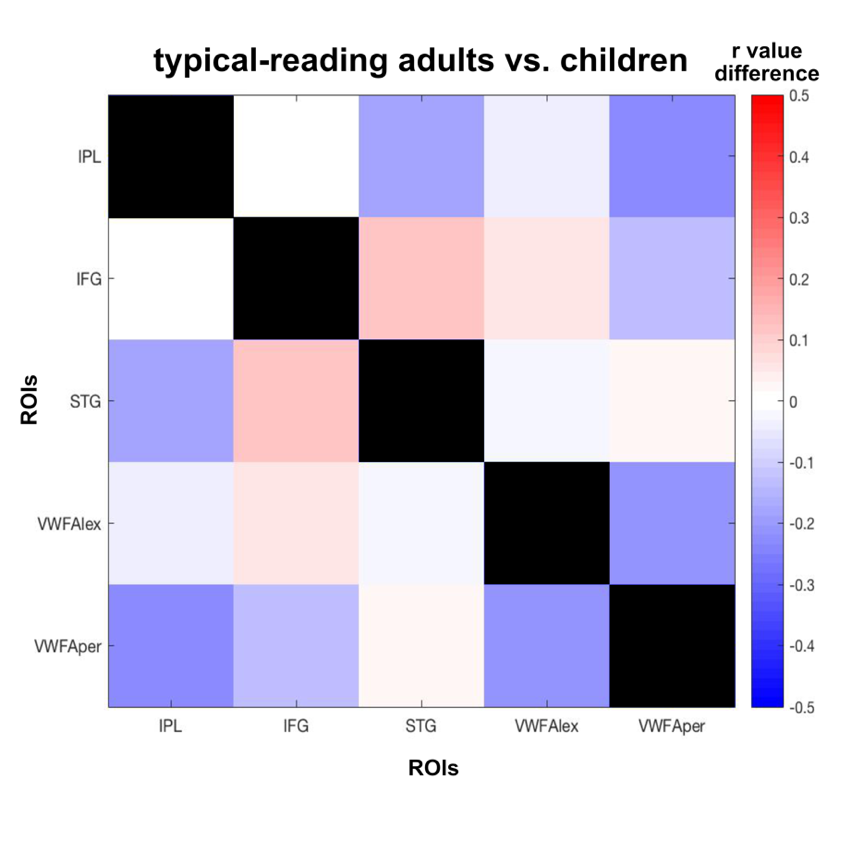
*

**Figure S1: Difference in structural covariance of reading-related brain regions between typical-reading adults and children.** We did not find a significant difference between typical-reading adults and children for structural covariance of reading-related brain regions. The color of each cell reflects the difference in structural covariance (typical-reading adults minus typical-reading children) of the corresponding column and row ROI.


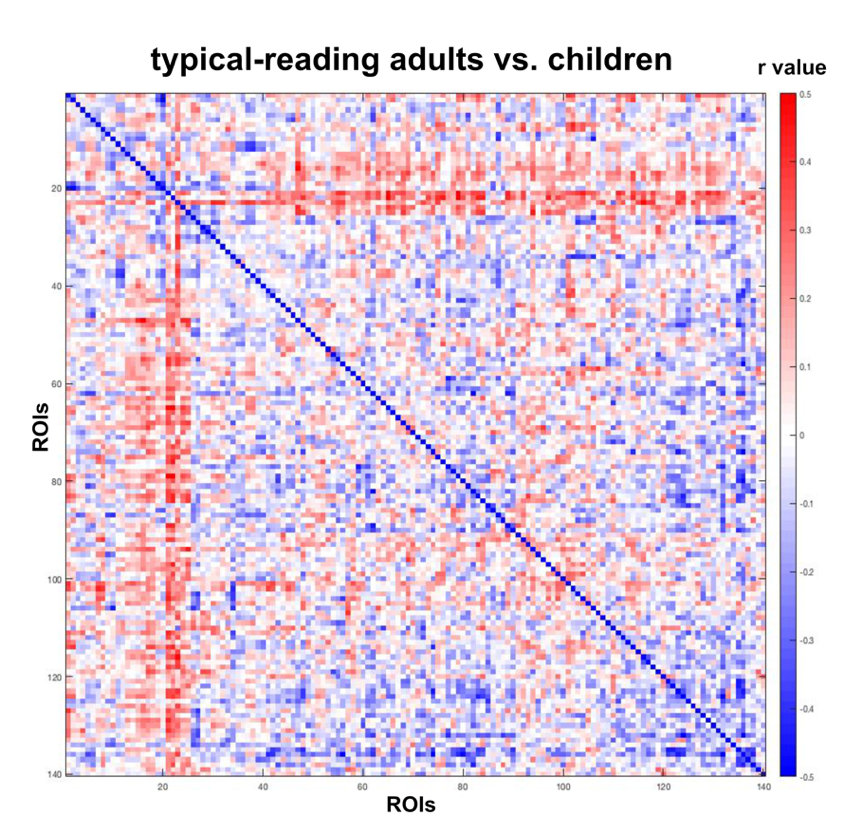


**Figure S2: Difference in structural covariance of 140 ROIs across the whole brain between typical-reading adults and children.** We did not find a significant difference between typical-reading children and adults for structural covariance of 140 ROIs across the whole brain. The color of each cell reflects the difference in structural covariance (typical-reading adults minus typical-reading children) of the corresponding column and row ROI.

*Structural covariance matrices in typical-reading children and children with poor reading skills*


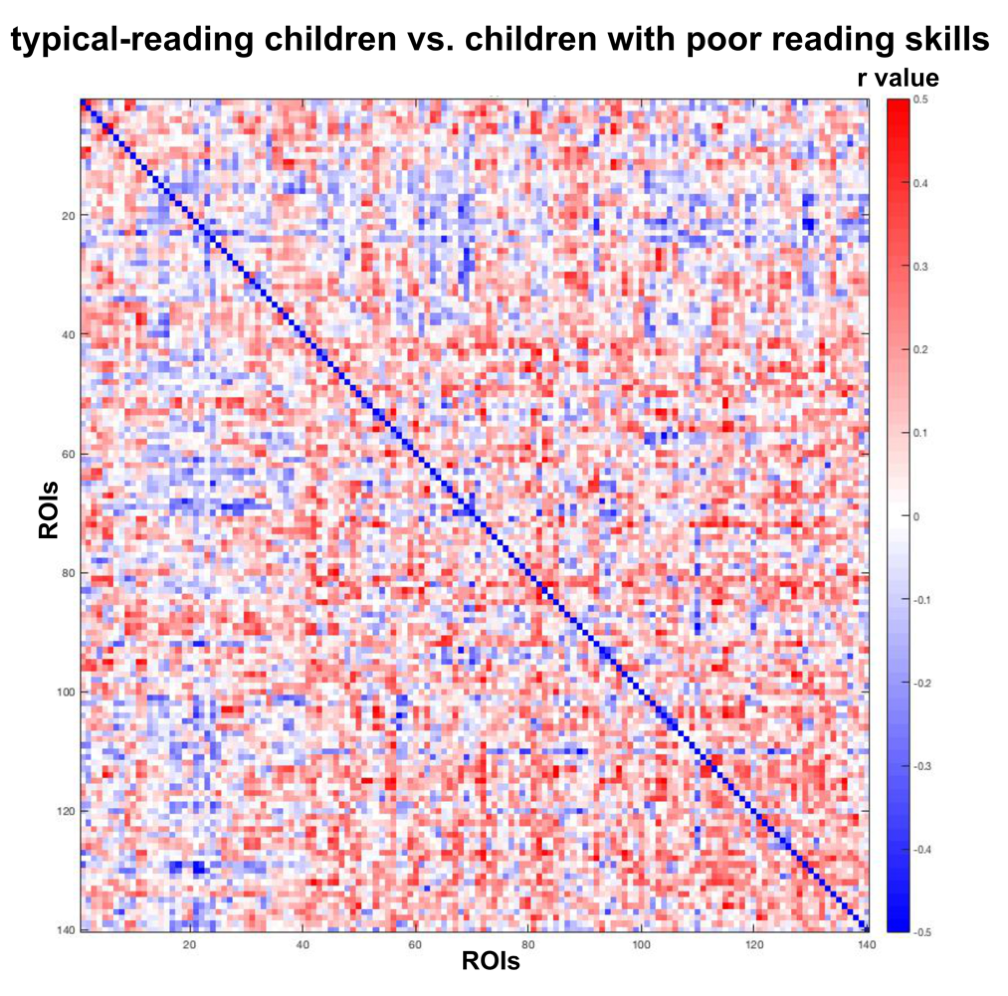


**Figure S3: Difference in structural covariance of 140 ROIs across the whole brain between typical-reading children and children with poor reading skills.** We did not find a significant difference between typical-reading children and children with poor reading skills for structural covariance of the whole brain. The color of each cell reflects the difference in structural covariance (typical-reading children minus children with poor reading skills) of the corresponding column and row ROI.

***Structural covariance matrices when using a smaller Gaussian kernel of 4mm full width at half maximum***

*Typical-reading adults versus typical-reading children*

When using a 4mm FWHM Gaussian smoothing kernel, typical-reading adults and typical-reading children did not differ significantly (p=0.18).


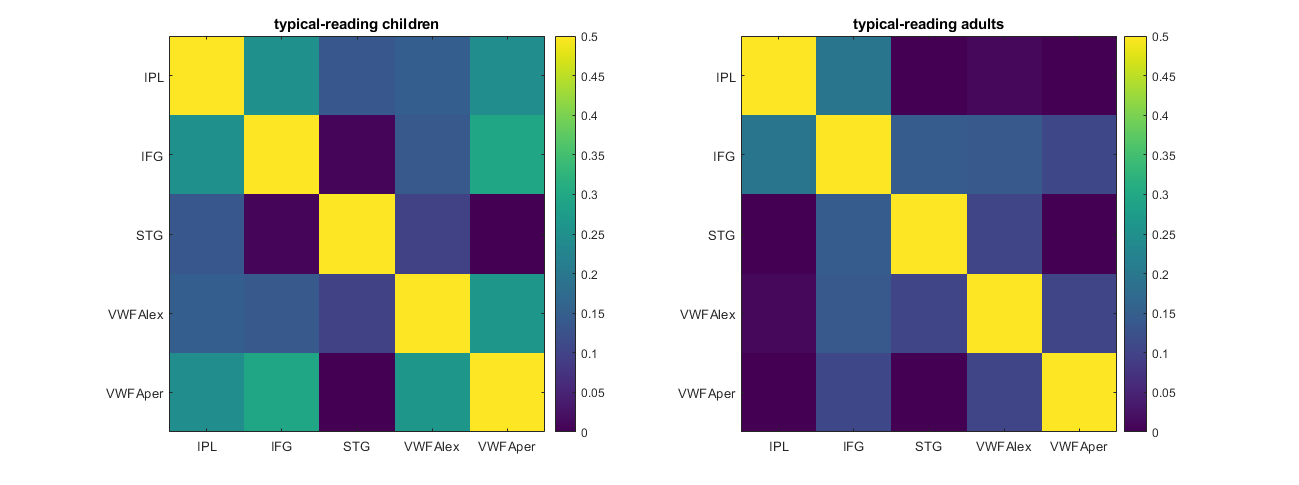


**Figure S4: Structural covariance of key regions of the reading network in typical-reading children (left) and adults (right) when smoothing with a 4mm full width at half maximum kernel.** We did not find a significant difference in the mean structural covariance of reading-related brain regions between typical-reading children and adults. The color of each cell reflects the structural covariance of the corresponding column and row ROI. Abbreviations: Inferior Parietal Lobule (IPL), Inferior Frontal Gyrus (IFG), Superior Temporal Gyrus (STG), lexical Visual Word Form Area (VWFA_lex), perceptual Visual Word Form Area (VWFA_per).


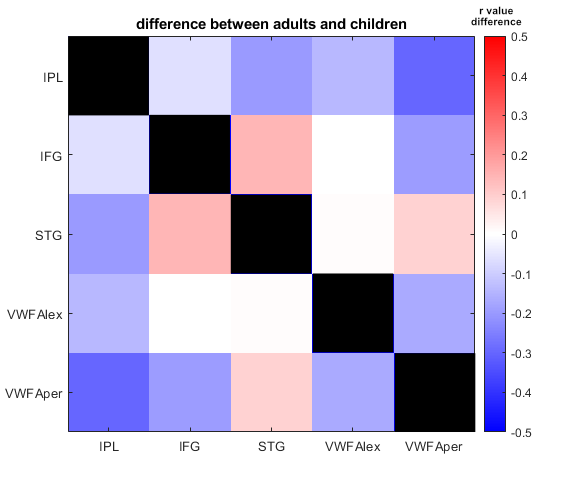


**Figure S5: Difference in structural covariance of reading-related brain regions between typical-reading adults and children when smoothing with a 4mm full width at half maximum kernel.** We did not find a significant difference between typical-reading adults and children for structural covariance of reading-related brain regions. The color of each cell reflects the difference in structural covariance (typical-reading adults minus typical-reading children) of the corresponding column and row ROI.

*Typical-reading children versus children with poor reading skills*

When using a 4mm FWHM Gaussian smoothing kernel, typical-reading children and children with poor reading skills showed a significant difference in their mean structural covariance (p=0.009).


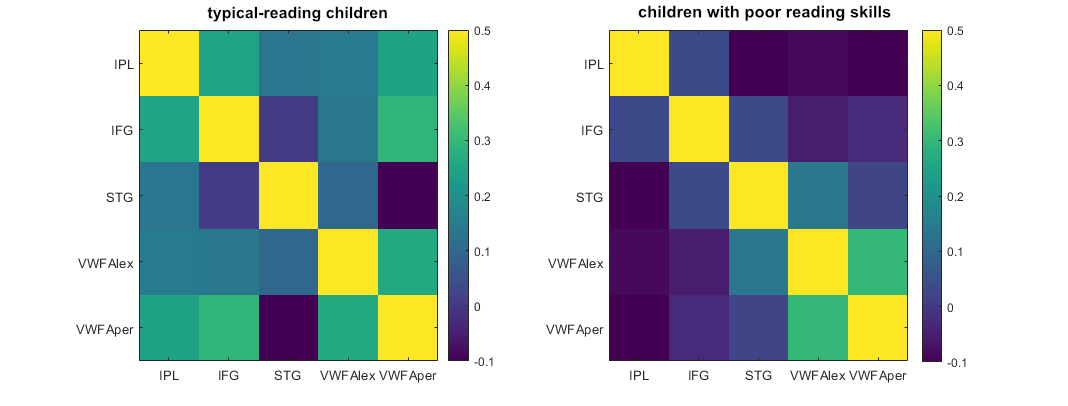


**Figure S6: Structural covariance of key regions of the reading network in typical-reading children (left) and children with poor reading skills (right) when smoothing with a 4mm full width at half maximum kernel.** On average, typical-reading children showed higher structural covariance between reading-related brain regions than children with poor reading skills (p=0.009). This was mainly driven by higher levels of structural covariance of the inferior parietal lobule (IPL) with other regions of the reading-network, such as between IPL-STG and between IPL-VWFA_per. The color of each cell reflects the structural covariance of the corresponding column and row ROI. Abbreviations: inferior parietal lobule (IPL), inferior frontal gyrus (IFG), superior temporal gyrus (STG), lexical visual word form area (VWFA_lex), perceptual visual word form area (VWFA_per).

To further investigate whether this difference was driven by the covariance between specific brain regions, we performed pairwise comparisons. These comparisons revealed significantly higher structural covariance for typical-reading children compared to children with poor reading skills between IPL-STG (p=0.04), IPL-VWFA_per (p=0.003), and IFG-VWFA_per (p=0.003). No significant differences were found for the IPL-IFG (p=0.16), IPL-VWFA_lex (p=0.13), IFG-STG (p=0.84), IFG-VWFA_lex (p=0.18), STG-VWFA_lex (p=0.79), STG-VWFA_per (p=0.39), and the VWFA_lex-VWFA_per (p=0.81). Only the IPL-VWFA_per and the IFG-VWFA_per structural covariance comparisons survived a multiple comparison correction using Benjamini & Hochberg false discovery rate (FDR) correction.


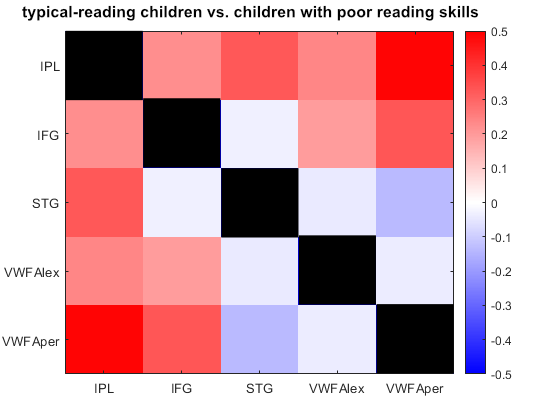


**Figure S7: Difference in structural covariance of reading-related brain regions between typical-reading children and children with poor reading skills.** We found a significant difference between typical-reading children and children with reading poor reading skills for structural covariance of reading-related brain regions (p=0.009). The color of each cell reflects the difference in structural covariance (typical-reading children minus children with poor reading skills) of the corresponding column and row ROI.

*Structural covariance matrices when excluding children with ADHD and dyscalculia*

When excluding children with ADHD (n=11) and dyscalculia (n=2), the results largely converged with the ones presented in the main text. We also observed a significant difference in overall structural covariance of reading-related brain regions (p=0.01). And pairwise comparisons also revealed a significant difference between children with typical (n=119) and poor (N= 59) reading skills for the IPL-STG (p=0.04), IPL-VWFA_per (0.004). No trend was found for IFG-VWFA_per (p=0.14), but rather for IPL-VWFA_lex (p=0.07). We found no significant difference between groups for the IPL-IFG (p=0.63), the IFG-STG (p=0.41), the IFG-VWFA_lex (p=0.34), IFG-VWFA_per (p=0.14), STG-VWFA_lex (p=0.26), STG-VWFA_per (p=0.19), and VWFA_lex-VWFA_per (p=0.73).

*
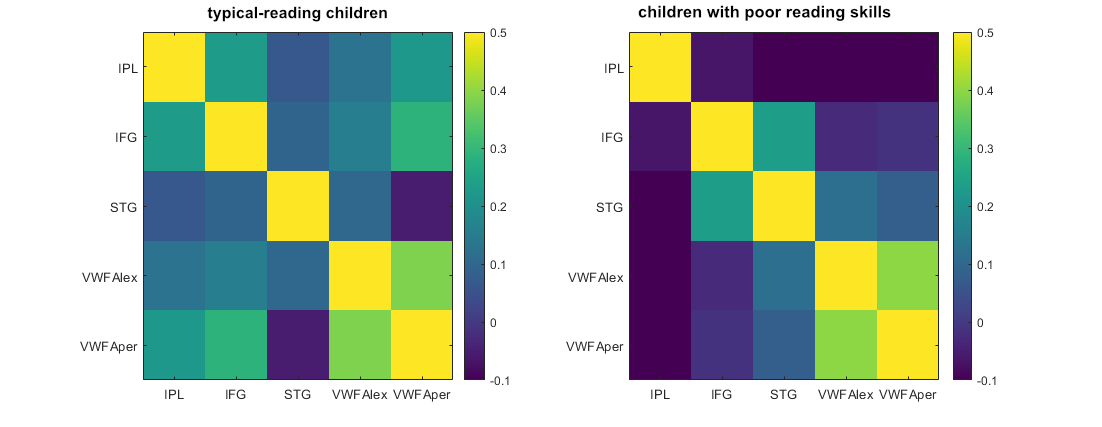
*

**Figure S8: Structural covariance of key regions of the reading network in typical-reading children and children with poor reading skills when children with ADHD and dyscalculia were excluded.** On average, typical-reading children showed higher structural covariance between reading-related brain regions than children with poor reading skills (p=0.01). This was mainly driven by higher levels of structural covariance of the inferior parietal lobule (IPL) with other regions of the reading-network, such as between IPL-STG and between IPL-VWFA_per. The color of each cell reflects the structural covariance of the corresponding column and row ROI. Abbreviations: inferior parietal lobule (IPL), inferior frontal gyrus (IFG), superior temporal gyrus (STG), lexical visual word form area (VWFA_lex), perceptual visual word form area (VWFA_per).


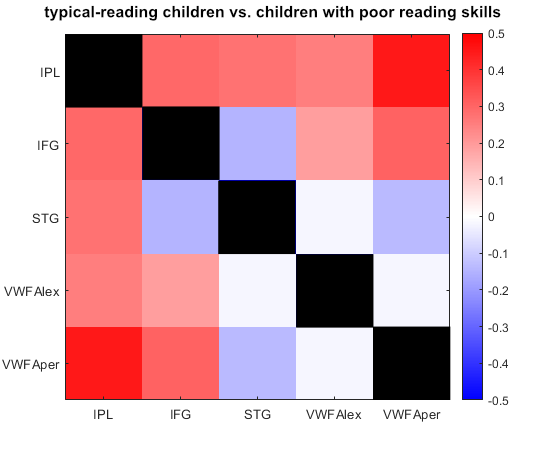


**Figure S9: Difference in structural covariance of reading-related brain regions between typical-reading children and children with poor reading skills when children with ADHD and dyscalculia were excluded.** We found a significant difference between typical-reading children and children with poor reading skills for structural covariance of reading-related brain regions (p=0.01). The color of each cell reflects the difference in structural covariance (typical-reading children minus children with poor reading skills) of the corresponding column and row ROI.

*Meta-analysis using Neurosynth and the keyword ‘reading’*

*
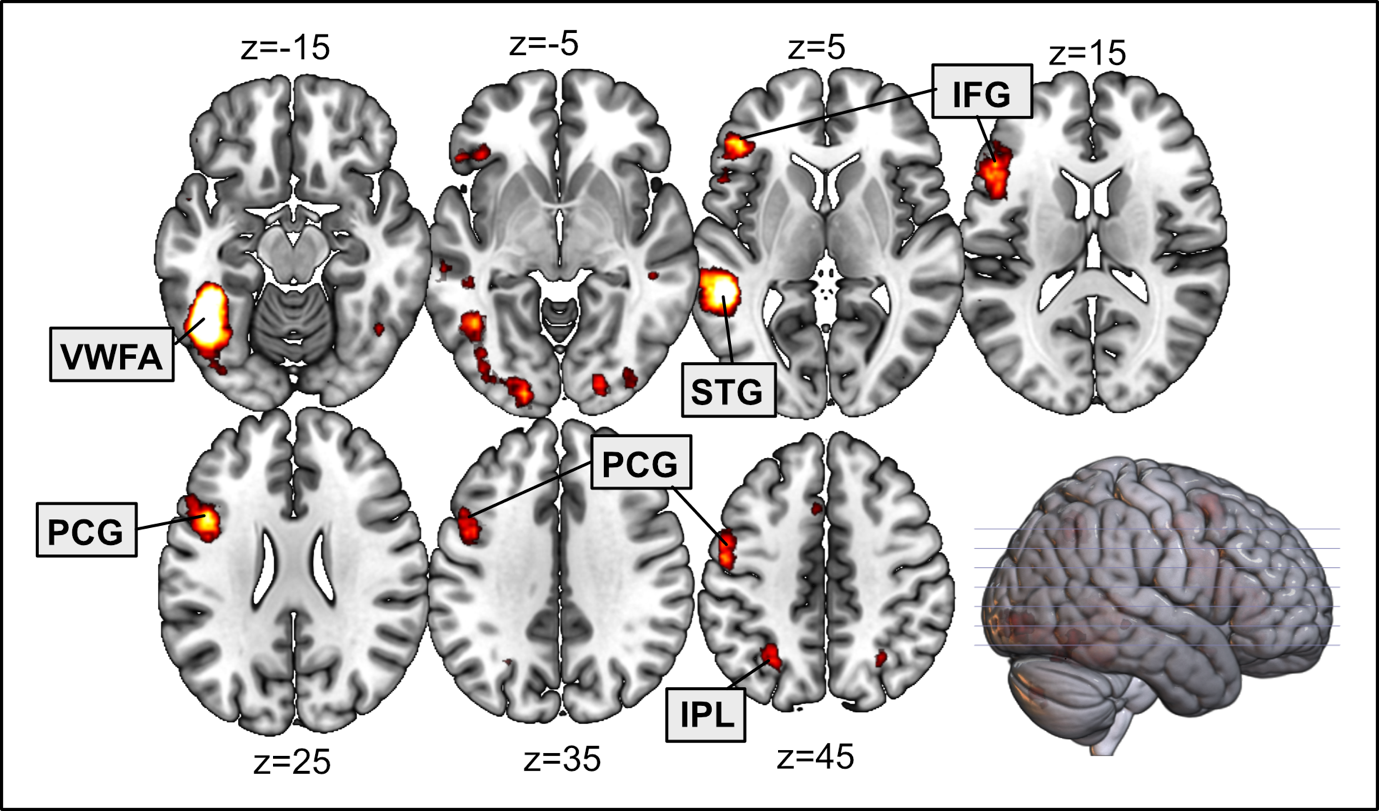
*

**Figure S10: Brain regions associated with the keyword ‘reading’.** Using Neurosynth, we performed a meta-analysis of the keyword ‘reading’. Based on 521 studies, the Neurosynth analysis revealed an association of the keyword ‘reading’ with activation in the visual word form area, superior temporal gyrus, inferior frontal gyrus, precentral gyrus, and inferior parietal lobule. The meta-analysis was performed by entering the keyword ‘reading’ and downloading the resulting brain map as a nifti file.
